# A Rare Case of Gastric Glomangioma Mimicking a Gastrointestinal Stromal Tumor (GIST)

**DOI:** 10.7759/cureus.98666

**Published:** 2025-12-07

**Authors:** Ahmet Yugruk, Altan Aydın, Orhan Semerci

**Affiliations:** 1 Department of General Surgery, Kanuni Training and Research Hospital - Trabzon Provincial Health Directorate, Republic of Turkey Ministry of Health, Trabzon, TUR; 2 Department of Pathology, Kanuni Training and Research Hospital - Trabzon Provincial Health Directorate, Republic of Turkey Ministry of Health, Trabzon, TUR

**Keywords:** diagnostic pitfalls, gastric benign neoplasm, gastric glomangioma, gastric glomus, gist, immunohistochemistry, mesenchymal tumor

## Abstract

Glomus tumors are rare mesenchymal neoplasms originating from modified smooth muscle cells involved in thermoregulation. Although typically located in peripheral soft tissues, their presence in the stomach is uncommon and may lead to diagnostic difficulties. This report presents a rare case of gastric glomangioma that clinically and radiologically mimicked a gastrointestinal stromal tumor. A 61-year-old woman presented in July 2024 with a three-month history of epigastric pain, nausea, and abdominal bloating. Upper gastrointestinal endoscopy revealed a submucosal lesion at the incisura angularis resembling a stromal tumor. Endoscopic ultrasonography demonstrated a well-circumscribed, submucosal, iso-hypoechoic mass measuring 19.5×11.3 mm. Due to persistent symptoms, surgical excision was performed, and the mass was completely removed with negative margins. Histopathological evaluation confirmed the diagnosis of a gastric glomus tumor. The postoperative course was uneventful, and no complications were observed during follow-up. This case emphasizes the importance of including glomus tumors in the differential diagnosis of subepithelial gastric lesions, particularly when imaging findings overlap with more common entities. Increased clinical awareness may improve preoperative diagnostic accuracy and support appropriate management strategies.

## Introduction

Glomus tumors are rare mesenchymal neoplasms arising from glomus bodies, which are specialized arteriovenous anastomoses involved in thermoregulation [1]. Although they are typically found in peripheral soft tissues, including the subungual regions, their occurrence in visceral organs, particularly within the gastrointestinal tract, is extremely rare. To date, fewer than 200 cases of gastric glomus tumors have been reported in the literature, underscoring their exceptional rarity. They account for approximately 1% of all glomus tumors and are most commonly located in the antrum [2,3]. Clinically, these tumors may present with nonspecific symptoms, such as epigastric pain, dyspepsia, nausea, vomiting, and upper gastrointestinal bleeding. Due to their submucosal localization and overlapping imaging characteristics, these tumors are frequently misdiagnosed as the more common gastrointestinal stromal tumors (GISTs) [4]. Distinguishing between these entities is crucial, as their management and prognosis differ significantly. Immunohistochemical profiling plays a pivotal role in diagnosis, as glomus tumors typically show positivity for smooth muscle actin (SMA) and negativity for cluster of differentiation 117 (CD117) and discovered on GIST-1 (DOG1) [5]. Complete surgical resection with negative margins remains the mainstay of treatment and serves both diagnostic and curative purposes. This study contributes to the limited literature on gastric glomangiomas and highlights the importance of considering these rare tumors in the differential diagnosis of subepithelial gastric lesions.

## Case presentation

A 61-year-old woman with class III obesity (body mass index: 43 kg/m², WHO classification) presented in July 2024 with a three-month history of persistent epigastric pain, nausea, and bloating. Her past medical history included type 2 diabetes mellitus, hypertension, hypercholesterolemia, and stage 2 chronic kidney disease secondary to diabetic nephropathy. On physical examination, no pathological findings were observed other than obesity. Initial laboratory evaluation revealed elevated serum gastrin and increased cancer antigen 19-9 (CA 19-9) levels, while renal function was decreased, consistent with her chronic kidney disease (Table 1).

**Table 1:** Laboratory findings at presentation. LDH: lactose dehydrogenase; CA 19-9: cancer antigen 19-9

Parameters	Values	Normal range
Hemoglobin (g/dL)	12.7	11-17
White blood cells (x10^3^/µL)	10.2	3.9-10.9
Platelets (x10^3^/µL)	216	100-380
eGFR (mL/min)	59	>90
Glucose (mg/dL)	184	74-100
Aspartate aminotransferase (IU/L)	16	<35
Alanine aminotransferase (IU/L)	22	<35
LDH (IU/L)	162	<248
Amylase (IU/L)	61	28-100
Serum gastrin (pg/mL)	520	13-115
CA 19-9 (U/mL)	85	0-37

**Figure 1:**
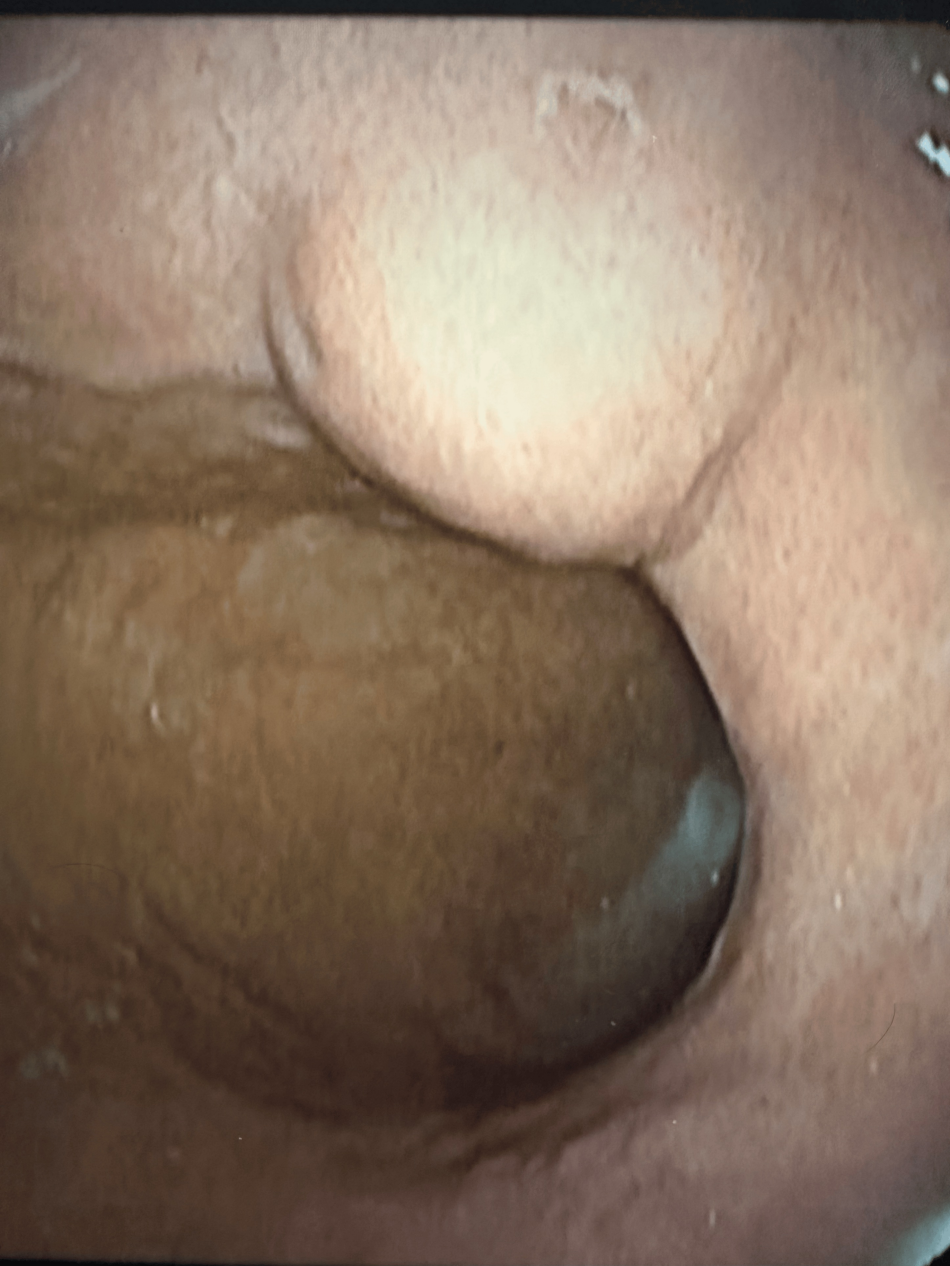
Endoscopic view of gastric glomangioma.

**Figure 2:**
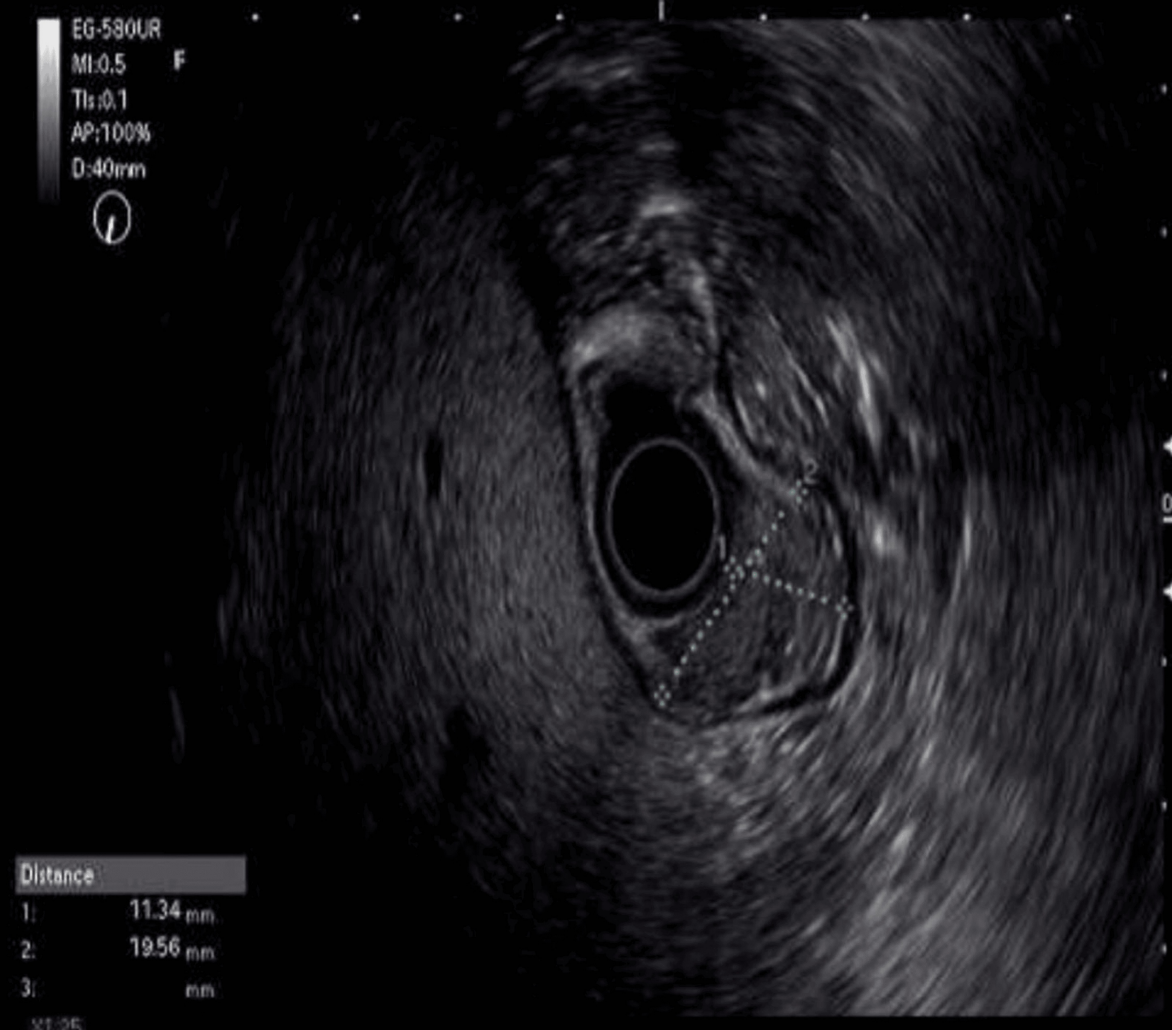
Endoscopic ultrasonography view of gastric glomangioma.

Upper gastrointestinal endoscopy demonstrated a 2 cm subepithelial lesion at the incisura angularis with intact overlying mucosa (Figure 1). Endoscopic ultrasonography revealed a well-circumscribed, iso-hypoechoic mass (19.5×11.3 mm) confined to the submucosa without invasion of the muscularis propria, with minimal cystic changes and poor vascularity (Figure 2). Abdominal computed tomography showed no evidence of metastatic disease.

**Figure 3:**
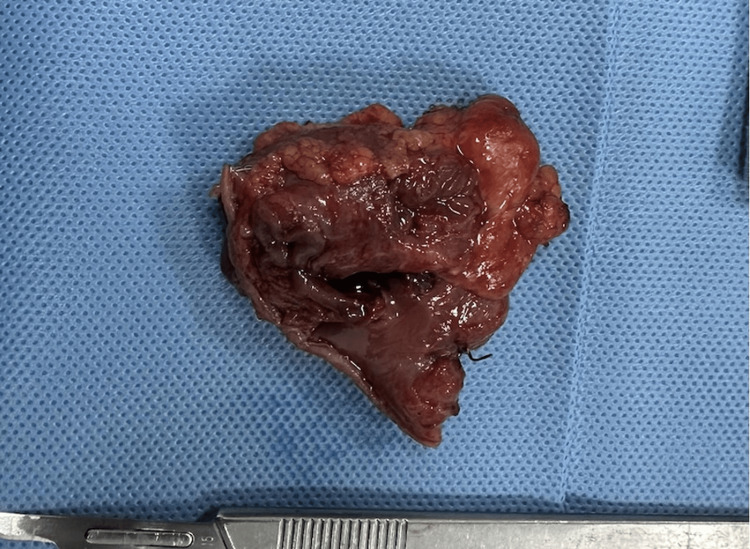
Surgical specimen material.

Given the persistence of symptoms and diagnostic uncertainty, exploratory laparotomy with wedge resection of the gastric lesion was performed. The resected specimen measured 3×3 cm and contained a well-defined 1.5×0.6 cm nodule, located 1 cm from the stapled margin, 0.2 cm from the mucosal margin, and 0.1 cm from the serosal surface (Figure 3).

**Figure 4:**
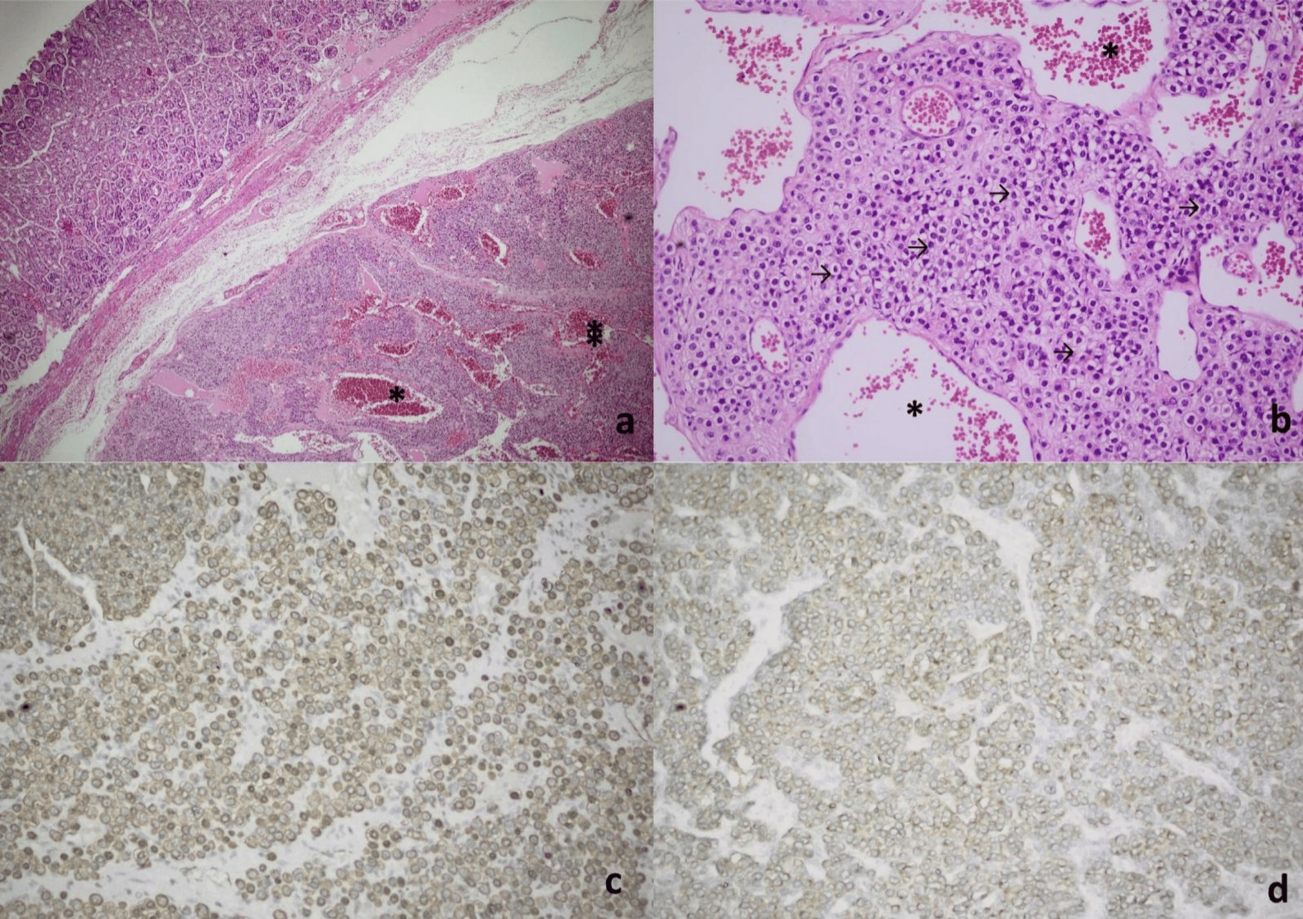
Histopathological and immunohistochemical features of gastric glomangioma. (a) Hematoxylin and eosin (H&E, ×40): a submucosal proliferative tumor lesion composed of small clusters of glomus cells surrounding dilated, erythrocyte-filled vessels (*), resembling cavernous hemangioma-like structures. (b) H&E (×100): the tumor consists of uniform, small, round glomus cells with monotonous round nuclei, vesicular chromatin, and amphophilic to lightly eosinophilic cytoplasm. These cells exhibited sharply defined borders (→) and were arranged in clusters surrounding dilated vascular structures (*). (c) Smooth muscle actin {SMA} immunohistochemistry {IHC} (×100): diffuse SMA positivity, consistent with glomus tumor phenotype. (d) Synaptophysin IHC (×100): strong synaptophysin expression, a potential diagnostic pitfall mimicking neuroendocrine tumors.

Histopathological examination revealed a well-circumscribed submucosal lesion composed of round glomus cells arranged around dilated, erythrocyte-filled vascular channels, resembling cavernous hemangioma-like structures. The tumor showed monotonous architecture with amphophilic to lightly eosinophilic cytoplasm and vesicular chromatin (Figures 4a-4d). Immunohistochemistry demonstrated positivity for smooth muscle actin (SMA), vimentin, and calponin, with negativity for cluster of differentiation 117 (CD117), Discovered on GIST-1 (DOG1), desmin, and S-100 (Table 2, Figures 4a-4d).

**Table 2:** Immunohistochemical examination. CD117 (c-KIT): cluster of differentiation 117; DOG1: Discovered on GIST-1; CD34: cluster of differentiation 34, SMA: smooth muscle actin; PanCK: pan-cytokeratin; CD45: cluster of differentiation 45; CD31: cluster of differentiation 31; HMB45: human melanoma black 45; SSTR2: somatostatin receptor type 2; GATA3: GATA binding protein 3

Markers	Results
CD117	Very focal weak positive
DOG1	Negative
CD34	Focal positive
Ki-67	1%
S100	Negative
SMA	Positive
Calponin	Positive
Caldesmon	Positive
Collagen type IV	Positive
Synaptophysin	Positive
Chromogranin	Negative
Desmin	Negative
PanCK	Negative
CD45	Negative
CD31	Negative
INSM1	Negative
CD56	Focal positive
CAM 5.2	Negative
SSTR2	Positive
GATA3	Negative

The final diagnosis of gastric glomangioma was established based on the combined histomorphology and immunohistochemical findings. The postoperative course was uneventful, and the patient was discharged on postoperative day five with regular follow-up. Clinical examinations were performed at one, three, six, nine, and 12 months, while abdominal CT imaging was obtained at six and 12 months of follow-up. At both six and 12 months, the patient remained symptom-free with no clinical or radiological evidence of recurrence.

## Discussion

Intramural mesenchymal tumors of the stomach include GISTs, various sarcomas, lipomas, leiomyomas, schwannomas, glomus tumors, hemangiomas, inflammatory fibroid polyps, inflammatory myofibroblastic tumors, and plexiform fibromyxomas. Among these, glomus tumors of the gastrointestinal tract (GIT) are rare, accounting for approximately 1% of GIT soft tissue tumors [2]. GISTs are the most common soft tissue tumors of the stomach [4]. Gastric glomus tumors are approximately 100 times less common than GISTs [6]. Gastric glomus tumors predominantly affect women between the ages of 50 and 60 years and typically present as solitary submucosal lesions localized in the antrum [5]. Symptoms are generally nonspecific and may include epigastric pain, bleeding, nausea, and vomiting.

Preoperative differentiation between glomus tumors and GIST remains challenging due to overlapping endoscopic and imaging findings, their deep submucosal location, and the lack of distinctive findings on CT scans [7,8]. Consequently, many gastric glomus tumors are identified incidentally during surgical intervention for presumed GISTs [2]. Immunohistochemical staining is essential for accurate diagnosis. Glomus tumors typically show strong SMA positivity and are negative for CD117 and DOG1, although rare cases may demonstrate focal or weak, non-specific CD117 staining that is not indicative of GIST. In our case, CD117 showed very focal weak positivity, consistent with this known but uncommon finding. DOG1 staining is increasingly recognized as a specific marker for diagnosing GIST [5]. Yoshida et al. reported that synaptophysin expression in gastric glomus tumors poses a significant diagnostic challenge, as evidenced by their report of a glomus tumor initially misdiagnosed as a neuroendocrine neoplasm due to strong synaptophysin immunoreactivity [3]. This paradoxical positivity, observed in 30-40% of cases according to Wang et al, requires careful interpretation alongside negative chromogranin and insulinoma-associated protein 1 results, to prevent misclassification [5]. The synaptophysin/chromogranin profile observed in this case is consistent with the findings of Vassiliou et al., highlighting that glomus tumors should be included in the differential diagnosis of synaptophysin-positive gastric submucosal lesions despite their non-neuroendocrine origin [7].

Although most gastric glomus tumors are benign, lesions exceeding 5 cm may raise concern for malignant potential [9]. The preferred treatment is complete surgical resection with negative margins, followed by long-term postoperative surveillance to monitor for recurrence [2].

## Conclusions

Gastric glomangioma, a vascular variant of glomus tumor, is a rare but clinically important neoplasm that can closely mimic more common subepithelial gastric tumors, particularly GIST. Accurate diagnosis depends primarily on histopathological and immunohistochemical examinations, as imaging findings are often nonspecific. This case highlights the importance of considering glomus tumors in the differential diagnosis of gastric lesions, especially when typical GIST immunomarkers are absent. Complete surgical resection with negative margins remains the treatment of choice, and long-term follow-up is recommended due to a low but possible risk of recurrence. Increasing awareness and reporting of such cases can enhance diagnostic accuracy and optimize patient management.
